# Trajectories of Sexual Risk Behaviors and the Associated Factors Among Young Men Who Have Sex With Men in China

**DOI:** 10.3389/fpubh.2022.854616

**Published:** 2022-03-21

**Authors:** Liqing Wei, Jiawei Tian, Menglan Guo, Biao Zhu, Qingqing Jiang, Bin Yu, Hong Yan

**Affiliations:** School of Public Health, Wuhan University, Wuhan, China

**Keywords:** YMSM, prospective cohort, HIV infection, trajectories, sexual risk behaviors

## Abstract

**Background:**

Young men who have sex with men (YMSM) are at high risk of HIV infection that accounts for an increasing proportion of new human immunodeficiency virus (HIV) infections in China. However, little is known about the trajectories of sexual risk behaviors in this population. The study aimed to investigate longitudinal patterns of sexual risk behaviors among YMSM in China.

**Methods:**

Study data were collected from a prospective cohort study among 460 YMSM from 2017 to 2020. Based on the predicted HIV infection risk scores, distinct sexual risk behaviors trajectories of YMSM were estimated and plotted using the group-based censored normal model to identify the predictors of trajectories change over time.

**Results:**

Three sexual risk behaviors trajectories were identified: a decreasing low-risk group (7.6%), an intermediate-risk group (67.4%), and an ascending high-risk group (25.0%).

Compared to the decreasing low-risk group, intermediate-risk group membership was associated with being from rural areas, current smoker and higher depressive symptoms; ascending high-risk group membership was associated with an education level of high school or lower, being from rural areas, younger age at sex debut with a man, current smoker, higher depressive symptoms and sexual minority stress.

**Conclusions:**

Sexual risk behaviors among YMSM changed over time within different trajectories. Identifying YMSM belonging to high-risk trajectories before HIV infection is vital for the intervention and may reduce HIV transmission.

## Introduction

According to the 2018 China acquired immunodeficiency syndrome (AIDS) response progress report, sexual transmission is the main mode of HIV infection in China, with sexual transmission accounting for 95.1% of new HIV infections (1). Men who have sex with men (MSM) accounted for 23% of newly diagnosed HIV cases in China in 2019, compared to only 2.5% in 2006 (2). Due to their active unprotected sexual practices, the incidence and prevalence of HIV in the MSM population are higher than any other high-risk group (e.g., female sex workers, drug users) (3). Data from the Chinese Center for Disease Control and Prevention (CDC) also showed that the temporal trend of HIV infection among MSM has been increasing in the past decades (4), especially in large cities (3). Therefore, identifying high-risk groups among MSM would be beneficial for the precise implementation of HIV interventions in China.

Young men who have sex with men (YMSM) are defined as MSM younger than 25 years. As a group whose capacity for self-regulation has not fully matured (5), they are more likely to engage in risky behaviors (6, 7). Data from CDC in the United States in 2015 showed that 92% of new HIV diagnoses among young American men aged 13–24 were from YMSM (8). Individuals' sexual risk behaviors are not static, especially in the early stages of one's physical and mental maturation, and may change over time in response to life events. However, knowledge on the trajectories of sexual risk behaviors in Chinese YMSM is quite limited. The study's main purpose was to identify the trajectories of sexual risk behaviors and the associated factors among Chinese YMSM.

Some risk screening models have been developed in western countries that use individual reported behaviors to evaluate the risk for HIV infection and identify high-risk individuals for interventions (9–12). These efforts have contributed to advances in the study of predictive sexual risk behaviors. However, the existing predictive models were all developed based on the western cultures, which may not be generalized to the MSM in the Asian population, including Chinese YMSM. In the Asian relatively conservative culture, MSM who maintain the interdependence of Asian cultural values is more likely to care about the health of their partners and therefore more likely to engage in safer sexual practices (13).

In this study, we used a revised version of the Delphi-based model developed for Chinese MSM to analyze data from a prospective cohort and predict the greatest risk of HIV infection among YMSM (14). The study has two aims: (1) to obtain distinct sexual risk behaviors trajectories among HIV-negative YMSM, (2) to identify behaviors and psychosocial factors associated with longitudinal trajectories of sexual risk behaviors.

## Materials and Methods

### Participants and Sampling

Data used in the study were derived from a prospective cohort study of YMSM from 2017 to 2020. The cohort aims to examine the epidemiology of YMSM, the psychosocial correlates of perceived stigma, and risk factors for the HIV infection process. The participants were recruited from three provincial capitals in central China, including Wuhan (capital city of Hubei Province), Changsha (capital city of Hunan Province), and Nanchang (capital city of Jiangxi Province). Participants were men who lived in the three cities and had had one or more male sexual partners in the past 6 months. At each survey site, we recruited participants through peer referrals, outreach events, networking, HIV testing with the assistance of MSM Non-profit Organizations (e.g., Qingcai of Changsha). Only MSM who agreed to participate and signed the informed consent were recruited. After the completion of the baseline survey, participants were asked to finish a follow-up survey every 6 months. The survey was delivered through a tablet at the survey site or through an online platform if the participants could not show up at the site. A final sample of 460 participants was recruited from the baseline during September 2017 and January 2018, and 214 participants completed the third wave of follow-up from October 2020 to December 2020.

### Measures

#### Sexual Risk Behaviors Score

This study used an individual HIV infection risk assessment tool for MSM established by Chinese scholars based on the Delphi method in 2017 (14). The tool used information from participants over the past 6 months to predict HIV infection risk score. Eight items were included in the assessment: (1) the number of male sexual partners, (2) HIV-positive partner status, (3) unprotected anal sex, (4) male-to-male commercial sex, (5) symptoms of sexually transmitted infections (STI), or diagnosis of STI (e.g., gonorrhea), (6) use of illicit drugs, (7) anal sex roles, (8) group sex. Since our data did not collect group sex information, we revised this tool accordingly (Table 1). The final predictors we used for inclusion in the risk score included: number of male sexual partners, HIV-positive partner status, unprotected anal sex (e.g., never, sometimes, always), male-to-male commercial sex (yes or no), symptoms of STI or diagnosis of STI (e.g., gonorrhea), use of illicit drugs (yes or no), anal sex roles [e.g., insertive anal intercourse (IAI), receptive anal intercourse (RAI), IAI, and RAI]. The score for each item was aggregated from the data collected at each visit, and the total score was the predicted YMSM HIV infection risk score. A higher predicted risk score for an individual means a higher probability of infection.

**Table 1:** Revised MSM HIV infection risk assessment tool.

**Sexual risk item**	**Reference risk score**
**Number of male sexual partners in the past 6 months**	
0~	1.00
2~	2.50
6~	3.50
≥10	4.50
**HIV-positive sexual partners in the past 6 months**	
No	1.00
Not sure	2.64
HIV-positive partners on antiretroviral therapy	1.76
HIV-positive partners not receiving antiretroviral therapy	5.27
**Unprotected anal sex in the past 6 months**	
No	1.00
Sometimes	3.32
Always	5.63
**Male-to-male commercial sex in the past 6 months**	
No	1.00
Yes	4.11
**Symptoms of STI or diagnosis of STI (e.g., gonorrhea) in the past 6 months**	
No	1.00
Yes	3.22
**Use of illicit drugs in the past 6 months**	
No	1.00
Yes	2.31
**Sex roles in the past 6 months**	
IAI only	1.00
RAI and IAI	2.34
RAI only	3.29

*IAI, insertive anal intercourse; RAI, receptive anal intercourse*.

#### HIV Testing

A rapid HIV antibody test (Runbio Biotechnology, China) was performed on each participant at each follow-up visit at the study site. For those who could not reach the site, we used their self-reported test results from the past 6 months.

#### Alcohol Use

Alcohol use was measured using the question “How often you have used alcohol (beer, red wine, yellow wine, white wine) in the past three months?,” with the options of “1 = none,” “2 = ever used 3 months ago,” “3 = once a month,” “4 = once a week,” “5 = almost every day.” We further classified them as none alcohol use (none), low/moderate alcohol use (ever used 3 months ago, once a month, once a week), and heavy alcohol use (almost every day).

#### Current Smoker

Tobacco use was measured using the question “Have you used tobacco (cigarettes, cigars, hookahs, chewing tobacco, e-cigarettes) in the last 30 days?” with answer options of “1 = yes,” “2 = no.” Those who answered “yes” to the question were classified as current smokers.

#### Depressive Symptoms

Depressive symptoms were assessed using the CESD (The Center for Epidemiologic Studies Depression Scale) scale (15). The CESD scale consists of 20 items, including 16 forward items (typical item: “I felt lonely”) and four reverse items (typical item: “I felt that I was just as good as other people”). A four-point Likert scale was employed to assess each item, ranging from zero to three, representing the duration of depression. The overall score for these 20 items is in the 0–60 range, with the more the scale value, the more severe depressive symptoms.

#### Sexual Minority Stigma

The scale of assessment of stigma toward homosexuality in China was employed to measure the sexual minority stigma (16). The scale consists of 10 items with two subscales perceived stigma (3 items, typical item: “How often have you had to pretend that you are not homosexual in order to be accepted?”), enact stigma (6 items, typical item: “How often have you lost your friends because of your homosexuality?”) and one item (“How often have you been made fun of or called names for being homosexual?”) that does not load onto either subscale. A four-point Likert scale was used to measure each item, ranging from 1 (never) to 4 (many times). An overall score for these ten items was calculated, with the more the value, the higher the level of sexual minority stigma.

#### Covariates

Baseline covariates for the study included age, ethnicity (Han/Minorities), education (high school or lower, college, master or higher), monthly income (in RMB: ≤1,000/1,001–3,000/3,001–6,000/>6,000), if a student (yes/no), residence (urban, rural), sexual orientation (homosexual, bisexual/undecided), age at sex debut with a man.

### Statistical Analysis

Descriptive analyses [e.g., mean, standard deviation (SD), frequency, percentage] were used to present the sample characteristics. Individual's HIV infection risk score was computed based on a risk assessment tool using information self-reported by the participant at each visit. Table 1 presented the scores for each survey question, and total scores were estimated with higher scores indicating a greater risk of HIV infection. We identified participants' HIV infection risk trajectories throughout the study period, using the PROC TRAJ procedure in SAS to perform a group-based censored normal model. This approach classified each participant's HIV infection risk score into “groups” at each visit, leading to different trajectories.

Briefly, first of all, we identified the optimal number of trajectory groups and calculated the probability of group membership and group membership assignment (Table 2). To determine the number of trajectory groups that best represented the heterogeneity of the groups in the data and best fitted the data, five criteria were used: (1) prior knowledge of HIV infection risk score trajectories, (2) Bayesian Information Criterion (BIC), (3) the average posterior probability of group membership (>0.7), (4) the significance of the curved profile of the trajectory group curve, and (5) group membership size (>5% for each group). With the number of groups determined, we changed the shape of the trajectory curve (i.e., linear, quadratic, and cubic) and chose the trajectory with the highest value of the Bayesian information criterion. These group trajectories were constructed based on data from those participants who participated at least twice during the study period.

**Table 2:** Fit statistics for group-based censored normal model with 2–5 classes.

**Number of classes**	**BIC**	**Posterior probability**
2	−3078.5	83.82%, 85.72%
3	−3068.4	82.51%, 81.18%, 81.59%
4	−3072.2	78.17%, 74.33%, 73.95%, 74.38%
5	−3077.9	75.09%, 62.80%, 62.63%, 70.95%, 78.36%

*BIC, bayesian information criterion*.

After we identified the optimal number of trajectory groups and shapes, the covariates of interest were incorporated into the trajectory model. Two types of covariates were considered for this analysis: time-invariant risk factors of trajectory group membership and time-varying covariates. These time-invariant risk factors include characteristics developed prior to the inception period of the trajectory that can be used to predict membership in a specific trajectory. Time-varying covariates were measured to estimate whether these covariates change the trajectory process of the trajectory group. The decreasing low-risk group was considered the reference group. All statistical analyses were completed using the commercial statistical software SAS version 9.4 (SAS Institute, Cary, NC, USA).

## Results

### Characteristics of the Study Sample

A total of 460 YMSM completed the baseline survey with 1,380 follow-up visits and, on average, three visits per participant. The mean length of follow-up was 23.5 months (14.5 months). The mean age at baseline was 21.0 years (2.1 years, see Table 3). The majority of participants were Han Chinese (91.5%). Among the total participants, 81.5% had a college education or higher, only 18.5% had a high school education or lower, 70.9% earned <3,000 RMB (equivalent to $480). More than half (56.5%) of participants were students. Almost 79.8% were homosexual in sexual orientation, and the mean age at sex debut with a man was 18.6 years (5.2 years), 34.6% never drank alcohol, 28.5% were current smokers. The mean sexual minority stress and depressive symptoms were 15.6 (SD 3.9), 18.4 (SD 11.8), respectively. Significant differences were found in the education, residence, sexual orientation, and sexual minority stress across three groups (all *p* < 0.05).

**Table 3:** Characteristics of HIV-negative participants at baseline visit.

**Characteristic**	**Decreasing low-risk (*n* = 35)**	**Intermediate-risk (*n* = 310)**	**Ascending high-risk (*n* = 115)**	**Total (*n* = 460)**	**χ^2^/F**	***P*-value**
Age, mean (SD)	21.1 (2.2)	21.0 (2.1)	21.0 (1.8)	21.0 (2.1)	0.29	0.75
Ethnicity, *n* (%)					2.39	0.30
Han	31 (88.6)	287 (92.6)	103 (89.6)	421 (91.5)		
Minorities	4 (11.4)	23 (7.4)	12 (10.4)	39 (8.5)		
Education, *n* (%)					18.25	0.001
High school or lower	2 (5.7)	53 (17.1)	30 (26.1)	85 (18.5)		
College	28 (80.0)	230 (74.2)	79 (68.7)	337 (73.3)		
Master or higher	5 (14.3)	27 (8.7)	6 (5.2)	38 (8.2)		
Monthly income, *n* (%)					5.51	0.48
0 ≤1,000	6 (17.1)	51 (16.5)	2 (17.4)	7 (16.7)		
1,001–3,000	17 (48.6)	171 (55.2)	6 (53.0)	249 (54.1)		
3,001–6,000	9 (25.7)	53 (17.1)	2 (22.6)	8 (19.1)		
>6,000	3 (8.6)	35 (11.3)	8 (7.0)	4 (10.0)		
Student, *n* (%)					5.31	0.07
Yes	24 (68.6)	177 (57.1)	5 (51.3)	2 (56.5)		
No	11 (31.4)	133 (42.9)	5 (48.7)	200 (43.5)		
Residence, *n* (%)					22.61	<0.001
Urban	25 (71.4)	168 (54.2)	48 (41.7)	241 (52.4)		
Rural	10 (28.6)	142 (45.8)	67 (58.3)	219 (47.6)		
Sexual orientation, *n* (%)					6.65	0.04
Homosexual	24 (68.6)	253 (81.6)	90 (78.3)	367 (79.8)		
Bisexual/undecided	11 (31.4)	57 (18.4)	25 (21.7)	93 (21.2)		
Age at sex debut with a man, mean (SD)	19.2 (2.1)	18.8 (6.2)	17.9 (2.1)	18.6 (5.2)	1.45	0.24
Alcohol use, *n* (%)					5.86	0.21
None	16 (36.4)	117 (36.4)	26 (27.4)	159 (34.6)		
Low/moderate	28 (63.6)	199 (62.0)	65 (68.4)	292 (63.5)		
Hazardous use	0 (0)	5 (1.6)	4 (4.2)	9 (2.0)		
Current Smoker					1.59	0.45
Yes	32 (72.7)	234 (72.9)	63 (66.3)	131 (28.5)		
No	12 (27.3)	87 (27.1)	32 (33.7)	329 (71.5)		
Sexual minority stress	16.3 (4.2)	15.2 (3.6)	16.5 (4.4)	15.6 (3.9)	5.22	0.006
Mean (SD)						
Depressive symptoms, mean (SD)	19.1 (12.5)	17.1 (11.3)	22.4 (12.1)	18.4 (11.8)	120.13	0.11

*SD, standard deviation*.

### Sexual Risk Behaviors Score

The sexual risk behaviors score estimation ranged between 7.00 and 20.03 across all the visits, with a mean risk score of 10.54 (SD 2.68). The area under the receiver operating characteristic (ROC) curve was 0.74 [95% confidence interval (CI), 0.65–0.83, cutoff score, 11.90].

### Sexual Risk Behaviors Trajectories

**Figure 1:**
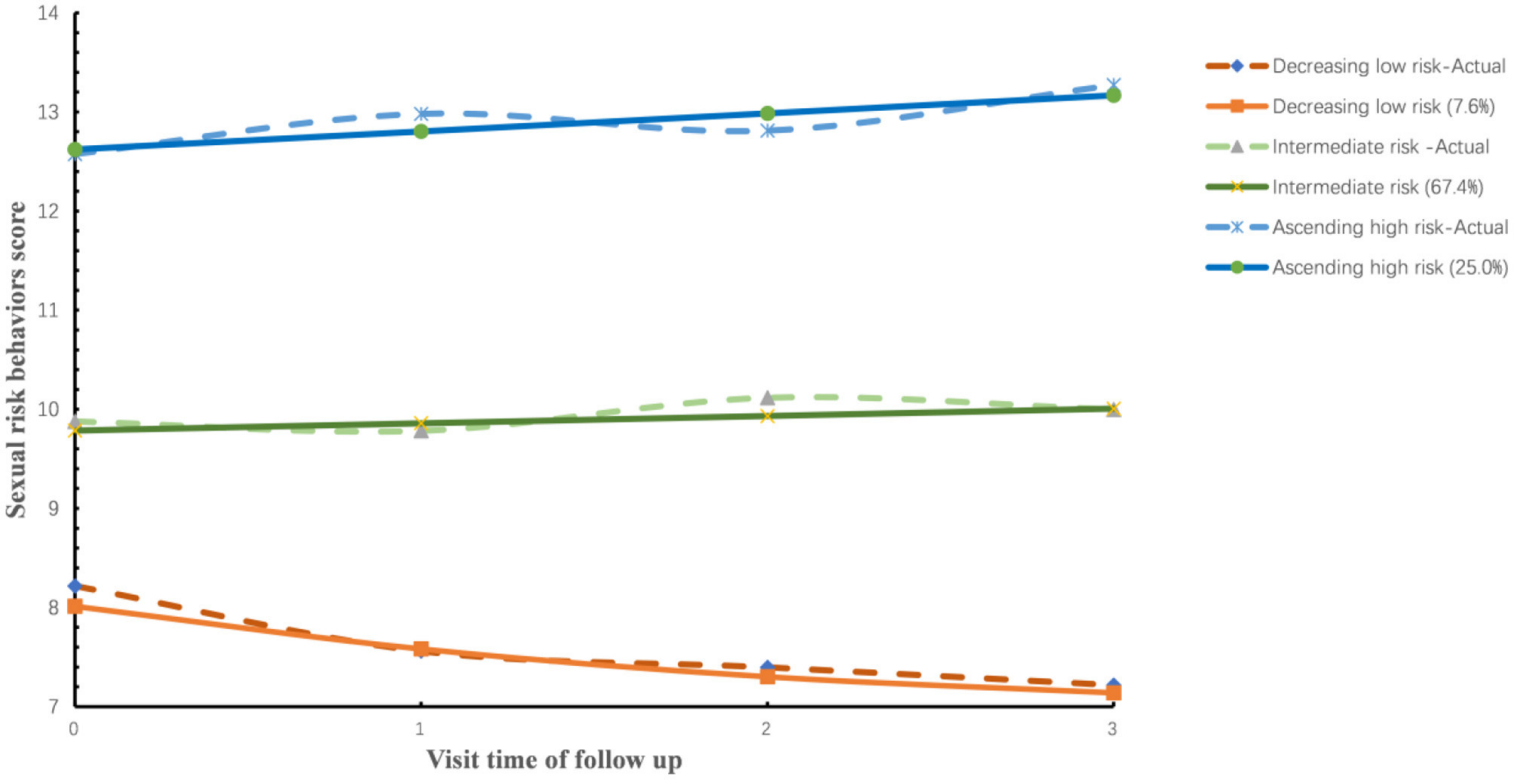
Sexual risk trajectories among 460 HIV-negative participants (2017–2020). The solid lines indicate the predicted probabilities of sexual risk behaviors score based on membership in one of the three sexual risk behaviors trajectory groups, whereas the dotted lines indicate the actual sexual risk behaviors score based on group membership.

Results in Figure 1 showed three sexual risk behaviors trajectory groups including (1) a decreasing low-risk group (*n* = 35, 7.6%), (2) an intermediate-risk group (*n* = 310, 67.4%), and (3) an ascending high-risk group (*n* = 115, 25.0%). Group membership in each group has an average posterior probability between 0.81 and 0.83, which indicates that our model has good classification quality.

Throughout the follow-up, 0.0% (0/35), 3.2% (10/310), and 7.8% (9/115) of decreasing low-risk group, intermediate-risk group, and ascending high-risk group were seroconverted, respectively. The HIV incidence rates in decreasing low-risk, intermediate-risk, and ascending high-risk groups were 0 per 100 person-year (PY), 1.74 per 100 PY, and 3.68 per 100 PY (*P* < 0.001), respectively.

### Multivariable Analysis of Time-Invariable and Time-Varying Factors of Sexual Risk Behaviors Trajectories Among 460 YMSM

Those who had completed a college or master's degree and higher (at their baseline visit), compared to those with a high school or lower were significantly decreased odds of membership in ascending high risk [Adjusted Odds Ratio, AOR = 0.21, 95% CI (0.05, 0.99); AOR = 0.12, 95% CI (0.03, 0.78); respectively] relative to those in decreasing low-risk group (Table 4). Compared to those who came from urban areas, MSM who came from rural areas had significantly increased odds of membership in intermediate-risk [AOR = 1.57, 95% CI (1.23, 3.61)] and ascending high risk [AOR = 3.20, 95% CI (1.25, 8.20)] relative to those in decreasing low-risk group. Age at sex debut with a man was associated with sexual risk behaviors trajectory membership. Those classified to ascending high-risk [AOR = 0.54, 95% CI (0.41, 0.72)] reported a younger age relative to decreasing low-risk group.

**Table 4:** Multivariable analysis of risk factors for sexual risk behaviors trajectory group membership among all men (*N* = 460).

**Characteristics**	**Decreasing low risk** **(*n* = 35) AOR (95% CI)**	**Intermediate risk (*n* = 310) AOR (95% CI)**	**Ascending high risk (*n* = 115) AOR (95% CI)**
Age	Reference	1.24 (0.90, 1.71)	1.57 (0.97, 2.19)
**Ethnicity (vs. Han Chinese)**			
Ethnic minorities	Reference	3.43 (0.83, 14.14)	1.69 (0.43, 6.64)
**Education (vs. High school or lower)**			
College	Reference	0.67 (0.12, 3.67)	0.21 (0.05, 0.99)^*^
Master or higher	Reference	0.94 (0.08, 11.14)	0.12 (0.03, 0.78)^*^
**Monthly income (vs**. **≤3,000)**			
≥3,001	Reference	1.02 (0.81, 1.30)	0.60 (0.40, 1.92)
**Student (vs. Yes)**			
No	Reference	1.56 (0.63, 3.85)	0.98 (0.35, 2.71)
**Residence (vs. Urban)**			
Rural	Reference	1.57 (1.23, 3.61)^*^	3.20 (1.25, 8.20)^**^
**Sexual orientation (vs. Homosexual)**			
Bisexual/undecided	Reference	0.61 (0.15, 1.33)	0.91 (0.24, 1.54)
Age at sex debut with a man	Reference	0.74 (0.56, 0.97)*	0.54 (0.41, 0.72)^**^
**Time-varying covariates influencing trajectories of sexual risk behaviors among all men (*****N*** **=** **460)**			
Heavy alcohol use	4.12 (0.74, 22.97)	1.31 (0.29, 6.03)	2.37 (0.32, 7.48)
Low/moderate alcohol use	0.75 (0.03, 1.27)	0.89 (0.56, 1.42)	0.82 (0.41, 1.67)
Current smoker	1.12 (0.73, 1.76)	1.71 (1.05, 2.78)^*^	1.75 (1.17, 3.56)*
Depressive symptoms	1.03 (0.98, 1.09)	1.04 (1.02, 1.07)^**^	1.06 (1.02, 1.09)^**^
Sexual minority stress	1.05 (0.92, 2.10)	1.04 (0.97, 1.11)	1.08 (1.00, 1.17)^*^

*AOR, adjusted odds ratio*.

* *P < 0.05*;

** *P < 0.01*.

Table 4 also shows the results of the impact of the covariates over time. Results showed that current smokers were associated with increasing sexual risk behaviors in intermediate-risk [AOR = 1.71, 95% CI (1.05, 2.78)] and ascending high-risk groups [AOR = 1.75, 95% CI (1.17, 3.56)]. Depressive symptoms were associated with increasing sexual risk behaviors in the intermediate-risk [AOR = 1.04, 95% CI (1.02, 1.07)] and the ascending high-risk groups [AOR = 1.06, 95% CI (1.02, 1.09)]. Sexual minority stress was associated with increasing sexual risk behaviors in ascending high-risk group [AOR = 1.08, 95% CI (1.00, 1.17)]. In addition, we did not find a statistically significant association between alcohol use over time and sexual risk behaviors trajectory group membership.

## Discussion

This study analyzed data from a four-year prospective cohort study of YMSM and applied the advanced group-based trajectory analysis to investigate the underlying varied groups of HIV infection risk. Study results revealed three typical trajectories of sexual risk behaviors throughout the study period among HIV-negative YMSM, including a decreasing low-risk group, an intermediate-risk group, and an ascending high-risk group. YMSM who were classified into these distinct groups also varied in their sexual risk behaviors and the risk of HIV infection, as well as factors of interest. Findings of the study provided important information to understand the dynamics of sexual risk behaviors among YMSM and devise and implement future effective intervention and prevention programs against HIV infection among YMSM in China.

Study results revealed three sexual risk behaviors trajectories with different risk of HIV infection. The ascending high-risk group showed the highest risk of HIV infection, with an incidence of HIV positive of 3.68 per 100 PY. Findings of the study also revealed an upward trend in sexual risk behaviors over time in the ascending high-risk group. That may be attributable to the lower mean age of sexual debut with a man than the intermediate and low-risk group. Individuals who had a sexual debut at an earlier age are more likely to be cognitively immature and more likely to be involved in high-risk behaviors, including high-risk sexual behaviors. Consistent with our findings, previous studies conducted in a USA cohort also found that the younger age of sexual debut was significantly associated with an increased risk of condomless anal sex (17). This finding may partly indicate that having sex with a man at an earlier age may have long-term effects on future sexual behaviors and increase the likelihood of engaging in risky sexual behaviors. Thus, more effective and informative sexual health education for adolescents is preferred, which may help YMSM delay the age of sex debut with a man and finally reduce their likelihood of engaging in sexual risk behaviors in the future.

While in the intermediate-risk group, we did not find a significant trend in the change of sexual risk behaviors. This group accounts for nearly 70% of participants, and their sexual risk behaviors were more likely to be relatively stable. However, it also means the previous interventions, including but not limited to health education programs, HIV testing, and consulting, were not effective and did not change YMSM's risk sexual behaviors. Thus, more theory-based, well-designed, and effective intervention programs are necessary to prevent and reduce high-risk sexual behaviors among this special population.

Study results indicated a decline in sexual risk behaviors among the decreasing low-risk group. The decline in risk behaviors may be associated with the increasing HIV concerns over time that were attributable to the advocacy interventions within the study sites (18). In addition, it had the highest proportion of people with university and higher education compared to the other two groups, and higher education may indicate higher self-awareness of HIV risk. Our speculation was supported by the fact that no one in this group became infected with HIV during the follow-up period. Ongoing interventions are needed for this population to reduce sexual risk behaviors, including health education programs, HIV testing, and counseling.

Our results suggest that several individual-level factors can characterize the different sexual risk behaviors trajectories for YMSM. We found that being rural at the baseline visit was associated with a higher likelihood of high-risk group trajectory. Other studies in China and the USA have shown that rural MSM had lower HIV testing rates, meaning they received less information on homosexual sex and had less knowledge of how to protect themselves from HIV infection (19, 20).

Study findings suggest that YMSM with a high school or lower education level were more likely to be classified into the ascending high-risk group. Higher educational attainment may be a protective factor against sexual risk behaviors (21). Consistent with our findings, previous studies have shown that highly educated MSM had lower HIV seroconversion rates (22). Another study in the USA MSM population indicated that higher levels of education were associated with increased frequency of HIV testing (23). Further, previous studies also suggest that MSM who are concerned about HIV status could reduce their sexual risk behaviors, such as reducing the likelihood of unprotected anal sex with a man whose HIV status was unknown (24).

High-risk trajectory membership has been linked to tobacco smoking status. In intermediate-risk and ascending high-risk groups, YMSM who were current smokers were associated with increasing sexual risk behaviors. Likewise, a study conducted among high school students in Hawaii, USA, showed that current smoker status increased the risk of HCV/HIV infection among MSM students compared to non-MSM students (25). A study conducted among college students in Guangxi, China, also showed that smoking was strongly associated with the risk of HIV infection among YMSM (26). Smoking is often associated with behaviors such as illicit drug use and depression, which are known predictors of non-adherence (27). Identifying this risk factor and providing behavioral interventions such as smoking cessation, especially at an early stage, can positively impact reducing HIV risk behaviors. Our study indicated that alcohol use was not associated with the trajectories of sexual risk behaviors. While many previous studies suggested that alcohol use, especially binge drinking, significantly increased the risk of HIV (28). The inconsistency may be attributed to the relatively lower prevalence of alcohol drinking behaviors in this young population in China (29). For example, 34.3% of our participants never drank alcohol, 42.5% drank only once or twice in the past three months, and only 2.2% drank heavily.

Another interesting finding in our study was that participants with higher depressive symptoms were associated with increasing sexual risk behaviors in intermediate-risk and ascending high-risk groups. Previous studies have found a positive association between depression and sexual risk behaviors (30, 31), including non-condom use, substance use, and multiple sexual partners. Our study also found an association between sexual minority stress and sexual risk behaviors trajectories. Studies found that sexual minority stress experienced by MSM can lead to engaging in risky sexual behaviors, and sexual minority stress may be a precursor to behavioral risk for HIV infection (32, 33). Comprehensive sexual risk reduction interventions, including improving mental health and addressing sexual minority stress, are critical to reducing sexual risk behaviors in this population. For example, individuals can reduce stigma and improve mental health by reducing social or emotional avoidance and promoting self-affirmation (34, 35).

The study has limitations. As a sexual minority, MSM are not accepted in the Chinese cultural context and are a hidden population. Therefore, we were unable to obtain samples by randomly sampling, and selection bias possibly existed due to the representativeness of the sample. Although we tried to include participants from different educational backgrounds in our recruitment process, most participants included in our sample are still highly educated and do not fully reflect the average level of education among Chinese MSM (36). Because perhaps the more educated groups have greater access to sexual minority organizations and HIV education and counseling opportunities, the risk of HIV infection among YMSM in our sample may be underestimated. In addition, our sample size is limited, and we may need to be more cautious in interpreting the results. This study used self-reported information from participants in the period prior to follow-up, and there may exist recall bias.

Despite these limitations, our study has strengths: (1) this is a prospective cohort among YMSM with the relatively long duration; (2) an HIV infection assessment tool suitable for the Chinese YMSM cohort was used; and (3) this study combined sexual risk behaviors score and group-based models for trajectory analysis, extending the use of this HIV risk assessment tool in cohort studies and validating the tool's utility.

## Conclusion

Study findings suggest that sexual risk behaviors in YMSM varied over time in different trajectories: decreasing low-risk group, intermediate-risk group, and ascending high-risk group. Our work enables the identification of individuals who fell into the highest risk trajectory based on different sexual risk score prior to HIV infection among YMSM. This information is critical for the timely delivery of targeted interventions and may reduce HIV transmission.

## Data Availability Statement

The raw data supporting the conclusions of this article will be made available by the corresponding author on reasonable request.

## Ethics Statement

The studies involving human participants were reviewed and approved by Medical Ethics Committee of Wuhan University. The patients/participants provided their written informed consent to participate in this study.

## Author Contributions

HY and LW designed the study. JT, MG, BZ, and QJ performed the field work. LW wrote the first draft of the manuscript. HY and BY revised the manuscript. All authors contributed to the article and approved the submitted version.

## Funding

This study was supported by the National Natural Science Foundation of China (Grant No. 81673196).

## Conflict of Interest

The authors declare that the research was conducted in the absence of any commercial or financial relationships that could be construed as a potential conflict of interest.

## Publisher's Note

All claims expressed in this article are solely those of the authors and do not necessarily represent those of their affiliated organizations, or those of the publisher, the editors and the reviewers. Any product that may be evaluated in this article, or claim that may be made by its manufacturer, is not guaranteed or endorsed by the publisher.
